# Microguards and micromessengers of the genome

**DOI:** 10.1038/hdy.2015.84

**Published:** 2015-09-30

**Authors:** D Green, T Dalmay, T Chapman

**Affiliations:** 1 Norwich Medical School, University of East Anglia, Norwich Research Park, Norwich, UK; 2 School of Biological Sciences, University of East Anglia, Norwich Research Park, Norwich, UK

## Abstract

The regulation of gene expression is of fundamental importance to maintain organismal function and integrity and requires a multifaceted and highly ordered sequence of events. The cyclic nature of gene expression is known as ‘transcription dynamics'. Disruption or perturbation of these dynamics can result in significant fitness costs arising from genome instability, accelerated ageing and disease. We review recent research that supports the idea that an important new role for small RNAs, particularly microRNAs (miRNAs), is in protecting the genome against short-term transcriptional fluctuations, in a process we term ‘microguarding'. An additional emerging role for miRNAs is as ‘micromessengers'—through alteration of gene expression in target cells to which they are trafficked within microvesicles. We describe the scant but emerging evidence that miRNAs can be moved between different cells, individuals and even species, to exert biologically significant responses. With these two new roles, miRNAs have the potential to protect against deleterious gene expression variation from perturbation and to themselves perturb the expression of genes in target cells. These interactions between cells will frequently be subject to conflicts of interest when they occur between unrelated cells that lack a coincidence of fitness interests. Hence, there is the potential for miRNAs to represent both a means to resolve conflicts of interest, as well as instigate them. We conclude by exploring this conflict hypothesis, by describing some of the initial evidence consistent with it and proposing new ideas for future research into this exciting topic.

## Introduction

### Small RNAs, RNA silencing and microRNA biogenesis

**Figure 1:**
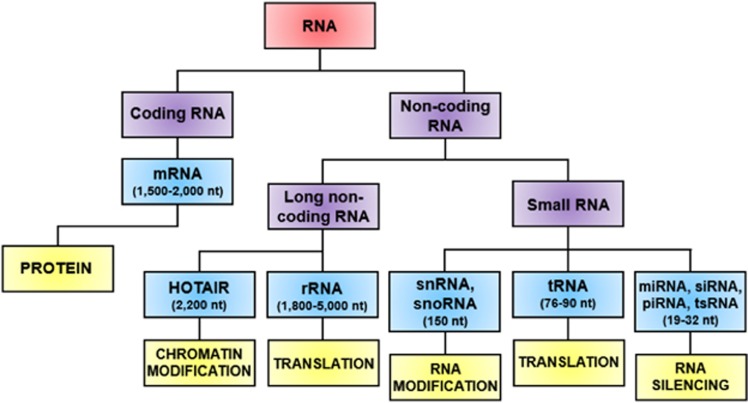
Eukaryotic RNA nomenclature and function. The small RNA family: small RNAs were initially defined as non-coding RNA molecules of 50–250 nucleotides (nt). However, with the discovery of a plethora of 19–32 nt non-coding families of RNAs over the last two decades, the term is now generally used to describe these ‘smaller' small non-coding RNAs (Perkel, 2013).

Small RNAs are a diverse set of functional, non-coding RNA molecules that are key regulators of expression for many genes in the genome via the process of gene silencing (Bartel and Chen, 2004; Hall and Dalmay, 2013). Initially, ‘small RNA' referred to any class of non-coding RNA molecule 50–250 nucleotides (nt) in length. However, over the last 10–15 years, the term is more often used to define ‘smaller' small RNAs of 19–32 nt, including small interfering RNAs, piwi-associated RNAs and the microRNAs (miRNAs) on which we focus in this review (Perkel, 2013; Figure 1). Small RNAs are produced via the processing of longer precursor RNAs. These precursors can be transcripts of small RNA genes, pre-messenger RNAs (from which introns are released during splicing and processed into small RNAs), tRNAs, small nucleolar RNAs and YRNAs. RNA polymerase II selectively transcribes mRNAs and most miRNA precursors, whereas other small RNAs are derived from housekeeping loci that are transcribed by RNA polymerase III. Small RNAs may be cleaved in a sequence- and structure-specific manner from parent RNAs such as YRNAs and tRNAs, with the latter tRNA-derived small RNAs then entering RNA-mediated gene silencing pathways (Kumar *et al.*, 2014). Research into small RNAs is currently proceeding at an astonishing rate, with ever-increasing knowledge of the discovery of the functions of the various classes of small RNAs as well as the pathways they utilise to alter gene regulation.

The huge interest in small RNAs is because of their roles in gene silencing through RNA-mediated mechanisms (Ghildiyal and Zamore, 2009). RNA silencing is an umbrella term for all small RNA-mediated inhibition of transcription, translation and deactivation of transposable elements. RNA silencing is widely regarded as a master controller of gene regulation. However, small RNAs are known to have important roles in an increasing variety of eukaryotic biological processes. For example, small RNAs have recently been discovered to have a role in transgenerational inheritance and epigenetic memory (Rechavi *et al.*, 2014).

**Figure 2:**
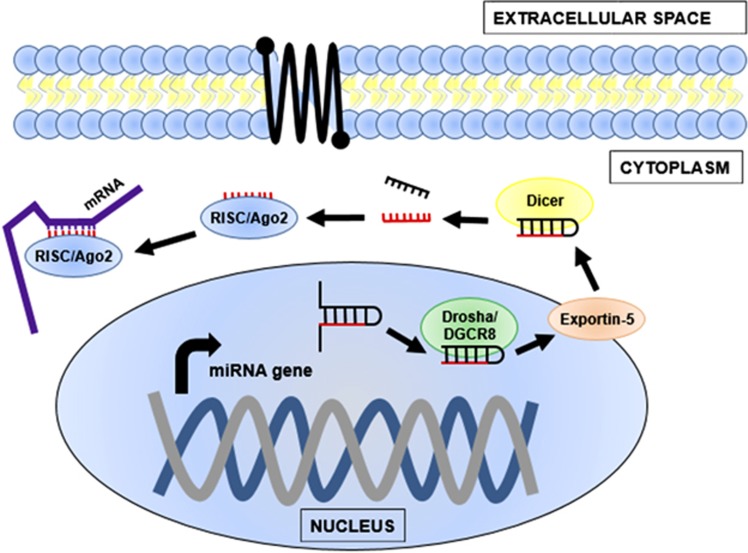
Biogenesis and mode of action of miRNAs. Pri-miRNA genes are transcribed from the genome by RNA polymerase II. The initial transcript is processed to become a shorter pre-miRNA by the microprocessor complex, a protein assembly of an RNase III enzyme known as Drosha and its RNA-binding partner, DGCR8 (also known as Pasha in fruit flies and worms). Exportin-5 facilitates the transport of the pre-miRNA into the cytoplasm. Here, the pre-miRNA undergoes further processing by an RNase III/helicase enzyme, Dicer, to become a ~22 bp miRNA duplex. The mature single-stranded miRNA is loaded into the RISC, which uses the miRNA sequence as a guide to bind complementary targets. Gene expression is modified by inhibition of ribosome loading, prevention of eukaryotic initiation factor function and mRNA cleavage.

We focus here on the most prominent and well-studied class of eukaryotic small RNAs, the miRNAs, which have diverse cellular roles but are best known for silencing and fine-tuning the expression of mRNA transcripts. miRNAs are evolutionarily conserved and can silence one to many hundreds of mRNA targets (Bartel and Chen, 2004; Kim *et al.*, 2009). In the canonical biogenesis pathway, miRNA genes are transcribed by RNA polymerase II (Figure 2). The pri-miRNA transcript is cleaved by Drosha/DGCR8 (or Drosha/Pasha in *Drosophila melanogaster* fruit flies). The subsequent ~70 nt pre-miRNA forms a characteristic hairpin secondary structure, which is vital for enabling export from the nucleus by RanGTP-dependant nuclear envelope-bound Exportin-5 (Filipowicz *et al.*, 2008; Lee *et al.*, 2004). Dicer, an RNase III/helicase multi-domain enzyme, processes the pre-miRNA into a ~22 bp miRNA-5p and miRNA-3p duplex. The pair is unwound with one mature miRNA strand selected by the Ago2/Piwi effector structure of the RNA-induced silencing complex. RNA-induced silencing complex uses the miRNA sequence as a guide for targeting complementary sequences in the mRNA 3'-untranslated region, also known as the miRNA recognition element. In animals, mRNA 5'-untranslated regions have recently been discovered to also contain miRNA recognition elements (Zhou and Rigoutsos, 2014). Accordingly, a gene is silenced or regulated via mRNA translational suppression, mRNA degradation or mRNA cleavage. The importance of miRNAs in gene regulation is illustrated by the evidence that they can regulate up to two-thirds of human genes (Hall and Dalmay, 2013). Gene regulation by miRNAs is of key importance in many fundamental biological processes such as cellular differentiation, proliferation, migration and apoptosis. Dysregulation of miRNA expression has been identified in many diseases and disorders (Dalmay, 2008; Swingler *et al.*, 2012). Therefore miRNAs are also promising biomarkers and potential treatment targets in genetic medicine.

In this review we focus on two new functions for miRNAs—their role as both guards against, and instigators of, gene expression variation. We first describe the evidence to support the idea that miRNAs can act as ‘microguards' of the genome and second that they can also be trafficked to, and alter gene expression in, target cells and hence function as ‘micromessengers'. We conclude by proposing that these two types of functions are likely to become the subject of conflicts of interest in the frequent situations in which interactions occur between cells with low relatedness (for example, different cell lineages, individuals or species). We review the evidence that is consistent with this hypothesis and propose further work to explore this exciting new possibility.

### miRNAs as molecular guards

Initially, much research focussed on understanding the ‘on/off' effects of miRNAs. Although this is crucial for understanding the functions and targets of miRNAs, it overlooks the role and key importance of miRNAs in the continual fine-tuning of gene expression to maintain organismal function (Kim *et al.*, 2013). Organisms have adapted to survive in the face of short-term variation in extrinsic (nutrition, temperature) and intrinsic (reproductive) stresses. However, continual bombardment from damaging exogenous factors may overwhelm stress protection responses and decrease fitness. New research proposes that this kind of ‘genetic inflammation' can be minimised by internal buffers in the form of miRNAs. Recent findings suggest that in organisms undergoing rapid change—for example, during development or in response to periodic external perturbations such as mating—miRNAs can switch from gene regulators to molecular guards (Fricke *et al.*, 2014; Kim *et al.*, 2013). In this latter role, miRNAs can buffer against abrupt fluctuations in mRNA transcription. This can be important to prevent deleterious effects of variation in mRNA transcript abundance and hence minimise genetic instability leading to a loss of fitness (see below). This ephemeral guarding mechanism is thought to be distinct from the sustained regulation of genes during longer-lasting, cyclical transcription dynamics (Hager *et al.*, 2009). Several examples of the microguarding phenomenon in different contexts are given below.

#### Microguarding to buffer fluctuations in gene expression during development

In the nematode worm *Caenorhabditis elegans* (and presumably many other organisms) thousands of transcripts either oscillate according to the different phases of larval development or show temporal gradients of increasing or decreasing expression (Kim *et al.*, 2013). The correct marshalling of these complex expression patterns is necessary for successful development. It has been shown that one mechanism by which a stable, increasing temporal gradient of an important developmental transcription factor known as *lin-14* can be achieved against this background of developmental oscillations is through the pulsatile expression of miRNA *lin-4* that targets it (Kim *et al.*, 2013). Synchronisation of the profile of expression of the miRNA against the background therefore results in a dampening of the oscillation of its target mRNA. That there has been natural selection to maintain this delicate balance and the microguarding potential of the miRNA is shown by the fact that a failure to correctly balance or integrate these signals can lead to fluctuations in mRNA transcription resulting in developmental arrest (Kim *et al.*, 2013). This example of a ‘miRNA-mediated incoherent feed-forward loop' reveals microguarding as a protective genetic layer against imbalances in post-embryonic developmental gene expression.

#### Microguarding to buffer against interactions with the opposite sex

Reproduction involves a complex series of choreographed events that must occur in the correct sequence in order for eggs to be produced, fertilised and laid/implanted. In invertebrates, many of the reproductive events that occur in females during and after mating are initiated by seminal fluid molecules transferred along with sperm during mating (Sirot *et al* 2014). In *D. melanogaster* there are >130 such proteins and peptides that cause striking changes to egg production, ovulation, female sexual receptivity, immune genes and sleep patterns (reviewed by Sirot *et al.*, 2014). Consistent with this, the receipt of seminal fluid proteins causes widespread, rapid gene expression (for example see Lawniczak and Begun 2004; McGraw *et al.*, 2004, 2008; Gioti *et al.*, 2012) and physiological changes (Sirot *et al.*, 2014) in females. The stimulation of this diverse suite of reproductive processes in females is not without potential cost and receipt of elevated levels of seminal fluid proteins causes reduced female lifespan and reproductive success (Chapman *et al* 1995; Wigby and Chapman, 2005). These effects are partly due to the actions of a specific seminal fluid protein known as the sex peptide (Chapman *et al.*, 2003b; Liu and Kubli 2003). The underlying genomic signatures of sex peptide receipt are evident in females in diverse changes in many mRNAs over time and in different body parts (Gioti *et al.*, 2012).

Post-mating responses initiated by one sex therefore lead to widespread changes in gene expression in the other (for example see Gioti *et al.*, 2012; Sirot *et al.*, 2014). There are many situations in which reproduction has the potential to cause unwanted fluctuations in gene expression leading to fitness loss for the focal individuals involved (for example see Wigby and Chapman, 2005). We therefore expect selection to buffer against the potentially deleterious mating-induced fluctuations in gene expression.

**Figure 3:**
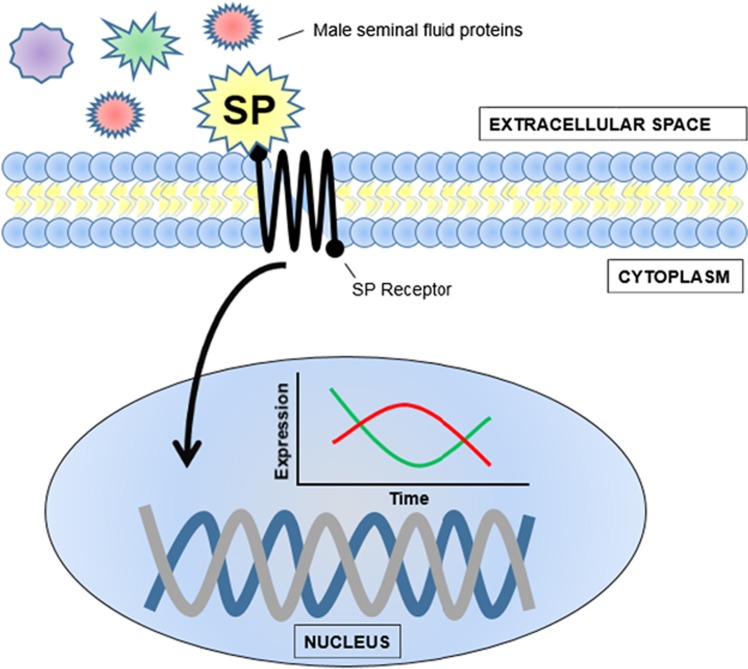
An example scheme for microguarding, using the fruit fly example. Sex peptide (SP), one of ~130 male seminal fluid proteins, is transferred into the female during mating and activates the SP receptor in the female genital tract and central nervous system. The activated SP receptor initiates intracellular signalling that alters the transcription of genes involved in behaviour, embryology, immunity and ageing. In this hypothetical model, deleterious fluctuations of mRNA expression in the female (red), facilitated by receipt of male ejaculate proteins, are potentially smoothed and dampened by synchronous expression of miRNAs by females (green) as a potential defence mechanism to reduce costs.

Consistent with this idea, the expression of mating-responsive miRNAs in females can protect against the sustained deleterious effects of reproduction-induced transcriptional changes (Fricke *et al.*, 2014; Figure 3). This conclusion was reached because females lacking specific miRNAs showed ‘unbuffered phenotypes'. For example, females that are hypomorphic for miR-279 (and/or potentially miR-996, see Sun *et al.*, 2015) and miR-317 showed higher reproductive output following mating than controls. Given that elevated reproductive output is accompanied by costs to females, the endogenous level of expression of these miRNAs may act to reduce reproductive output and hence minimise any potential costs. These miRNAs appear to function in this context to block against increased short-term, mating-induced increases in reproductive output, in order to maintain female fitness and extend reproduction over a longer time scale. Similarly, females lacking miR-279 (and/or miR-996, Sun *et al.*, 2015) and miR-317 also tended to re-mate more readily than controls, which suggests that the normal wild-type level of expression could serve to limit over-frequent and costly matings (Chapman *et al.*, 1995, 2003a). By expressing these miRNAs the female can potentially safeguard her genome against deleterious gene expression fluctuations arising from reproduction-related energy expenditure. The results were also consistent with the idea that continual exposure to the exogenous stress of mating with males is more costly to females when miRNA levels were reduced. Specifically, females lacking the miR-278 locus had significantly reduced lifespan upon continual exposure to males than controls. Therefore, the wild-type level of miR-278 was apparently protective against female loss of life. Microguarding in these examples appears to modify genomic responses to exogenous reproductive signals to prevent genome instability, accelerated ageing and an early demise.

#### Microguarding against the effects of infection

The deleterious consequences for host fitness of perturbation of gene expression arising from infection are widespread. We would again expect selection for microguards to reduce the deleterious impact of such fluctuations. Such an example comes from the study of human infection. Inflammation is a carefully choreographed immune response to a site of contagion, with toxic molecules and chemokines released into the microenvironment. Although this is lethal to invading pathogens, it is also harmful to surrounding healthy tissue. Therefore, inflammatory responses are usually short lived. It is well-recognised that chronic inflammation can increase the risk of age-related diseases such as cancer and musculoskeletal disorders. Under sustained inflammation, mechanisms that limit and protect the body against the deleterious side effects may break down. In an experiment on human cells, a pro-inflammatory environment was found to induce the increased expression of miR-155 (Tili *et al.*, 2011). A target transcript of this miRNA is the serine/threonine-specific protein kinase *WEE1*, a cell cycle inhibitor and tumour suppressor gene. Other miR-155 targets included DNA mismatch-repair genes: creating a downward spiral of further mutation rates in the purine salvage enzyme hypoxanthine-guanine phosphoribosyltransferase. External interference to microguarding is analogous to chronic inflammation. A re-balancing of this system may offer a new approach for studying and treating such disorders.

The examples listed above may represent just the tip of the iceberg and much more research is clearly needed to discover how widespread the phenomenon of microguarding might be. We need further fitness tests of the consequences of the lack of buffering functions across many different contexts and to better understand the selective forces that will promote microguarding function. However, the examples show proof of principle that the absence or over-abundance of microguarding may result in significant fitness costs. Hence there are significant potential benefits associated with the ability to microguard in response to potentially diverse sources of perturbation.

### miRNAs as molecular messengers with the potential to alter gene expression

Cell-to-cell communication can occur via direct contact or by the actions of molecules that are secreted into the extracellular space to reach local or distant targets. There has been huge recent interest in the role of miRNAs in extracellular communication. The big surprise has been the discovery that extracellular miRNAs can remain stable, and be transported, if they are packaged in microvesicles (MVs). Hence miRNAs can, in principle, retain their biological functionality to regulate gene expression in cells distant from their site of synthesis (Valadi *et al.* 2007). This has given rise to a new and exciting hypothesis that miRNAs may mediate an important new mechanism of cell–cell communication (Valadi *et al.* 2007; Wang *et al.*, 2010). Hence, along with the potential for miRNAs to function as microguards as described above, that they may also be transported within and between individuals as micromessengers.

The initial interest in miRNAs as micromessengers was stimulated by the finding that miRNAs can be found at appreciable levels within extracellular MVs (Valadi *et al.*, 2007). miRNA-containing MVs are now known to found in many different biofluids such as plasma, breast milk and semen (Vojtech *et al.*, 2014) and can be released by many cell types (Lotvall and Valadi, 2007). Small RNA and mRNA trafficking first achieved widespread recognition in plants (Yoo *et al.*, 2004; Marin-Gonzalez and Suarez-Lopez, 2012; Parent *et al.*, 2012) and has now also been recognised as potentially widespread in animal cells (Valadi *et al.*, 2007).

However, in order for miRNAs to be molecular messengers with the capacity to be trafficked to remote sites in animals, suitable transport vehicles are required for stability and transport. For example, RNAs need to be protected against degradation by RNAses in the extracellular environment (Chen *et al* 2008; Mitchell *et al.* 2008). Valadi *et al.* (2007) first reported that miRNAs could be packed into MVs and also demonstrated proof of principle that MV-associated RNA could be transferred into recipient cells. miRNAs, as well as mRNAs, proteins and DNA can be found within a variety of different types of MVs that are released from cells into the extracellular environment (Mathivanan *et al.*, 2010; reviewed by Weilner *et al.*, 2013).

MVs are diverse and are generally classified on the basis of size, shape, composition and origin. The classification of MV types remains complex—however, there are generally thought to be three types: ectosomes, exosomes and apoptotic bodies. Ectosomes bud off from the plasma membrane, exosomes are much smaller MVs and formed from vesicles within the cell that can then fuse with the plasma membrane and apoptotic bodies pinch off from the plasma membrane. Though the characteristics of the different types vary, all of them have been found to have the capacity to contain miRNAs once they emerge into the extracellular environment (Weilner *et al.*, 2013). miRNAs can certainly be found inside MVs, but may also adhere to the MV membrane. What is not yet clear however, is whether the concentrations of miRNAs within any/some/all of the various types of MVs are always sufficient to effect gene silencing in target cells (Skog *et al.*, 2008; Chevillet *et al.*, 2014). That said, non-conventional roles for miRNAs might be effected at lower concentrations, a possibility that requires further testing. In this review we refer to MVs as a whole, but recognise that there is huge complexity within this class of biological transport vehicles, whose significance in this context is far from being understood.

For miRNAs to be trafficked within MVs and perform biologically relevant roles when reaching their destinations, three conditions need to be fulfilled:

#### First: different populations of cells or tissues can produce MVs containing different and characteristic sets of miRNAs

There is growing evidence that the packaging of miRNAs into MVs can indeed exhibit tissue specificity (Braicu *et al.*, 2015). For example, evidence to support the idea of a specific, controlled packaging mechanism for miRNAs has been reported in liver cells by Kogure *et al.* (2011). Similarly, MVs may also contain a non-random subset of miRNAs that occur within the parent cells as observed by Kogure *et al.* (2011), supporting the idea of specific packaging mechanisms. Many mammalian cells in culture retain the capacity to export miRNAs into the surrounding environment, which may explain the observation of high concentrations of miRNAs in fluids such as plasma (Wang *et al.*, 2010). Though much more work is needed, in principle there is accumulating evidence that cells have the potential to package specific sets of miRNAs into MVs.

#### Second: MVs are mobile and deliver their payload to potential target cells

**Figure 4:**
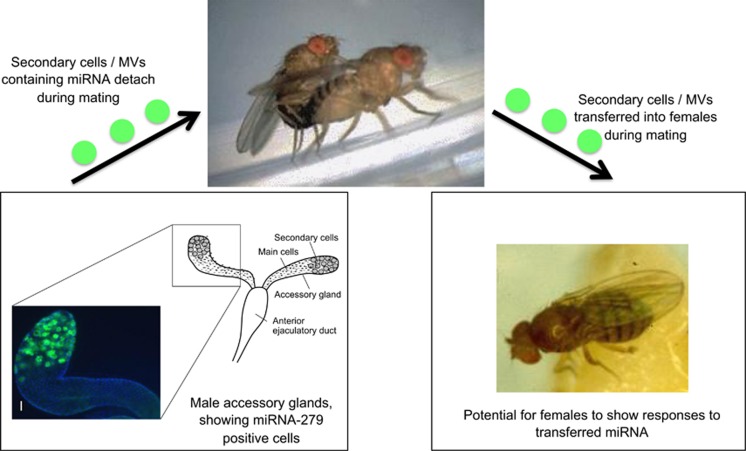
A hypothetical model for miRNA transfer between the sexes. Here, miR-279, which is implicated in microguarding against the costs of gene expression variation in females arising from sexual conflict (Fricke *et al.*, 2014), has the potential to be transferred to females from males during mating. miR-279 is expressed in the secondary cells of the *D. melanogaster* male accessory gland (Green *et al.*, unpublished data). The inset shows a confocal micrograph of the tip of an accessory gland of a 1 day-old male reproductive system, in a transgenic line in which the expression of miR-279 is tagged to a GFP reporter (*miR-279-GAL4; UAS-GFP*; Cayirlioglu *et al.*, 2008). The bi-nucleate arrangement of the nuclei in the accessory gland main cells is evident by DAPI staining (blue). Scale bar, 50 μM. These secondary cells can detach and transfer across to females during mating (Leiblich *et al.*, 2012). The secondary cells also release microvesicles (MVs) that are similarly transferred during mating and that can interact with female tissues and alter female post mating receptivity (Corrigan *et al.*, 2014). This system therefore offers a potential model for miRNA trafficking between the sexes, in a process likely to be shaped by sexual conflict.

RNAs can be transferred within MVs between immune mast cells in mouse and from mouse to human mast cells (Valadi *et al.*, 2007). miRNA transfer in MVs from T cells to antigen presenting cells has also been shown (Mittlebrunn *et al.*, 2011). In a different cellular context there is evidence that MVs released by male reproductive tissues in humans and in *D. melanogaster* fuse to sperm (Corrigan *et al.*, 2014) and therefore have the potential to be transported to very specific places in the female—although it is not yet known whether these MVs contain miRNAs. To expand: in *D. melanogaster*, one of the two main cell types of the adult male accessory gland, the secondary cells (Figure 4), have been observed to secrete MVs (Corrigan *et al.* 2014), which, after mating fuse with sperm and can interact with the reproductive tract of the female. Indeed the prevention of the release of these MVs altered the normal inhibition of female sexual receptivity following mating.

#### Finally: the miRNAs delivered into cells can affect the expression of specific target genes

The final step is that miRNAs trafficked to specific cells in MVs need to initiate specific cell responses, that is, suppress the activity of specific mRNAs targeted by the miRNAs within the MVs. It is thought that miRNAs have the capacity to be delivered into target cells via fusion of MVs with the target plasma cell membrane or by endocytosis of MVs (reviewed in Weilner *et al.*, 2013). Evidence that this can occur in principle comes mostly from *in vitro* experiments, particularly in the study of cancer and immune biology. The transfer of miRNA-bearing MVs between cell types has the potential to suppress target gene expression in the recipient cells (for example see Buck *et al.*, 2014; Pegtel *et al.*, 2014; Squadrito *et al.*, 2014). For example, Skog *et al.* (2008) incorporated a reporter into MVs and showed that the RNAs trafficked within MVs (exosomes) from glioblastoma cancer cells into normal cells can be translated. The authors suggested that this may promote the formation of a tumourigenic environment (Skog *et al.*, 2008). Another example occurs in liver cells in which MVs contain a highly enriched fraction of miRNAs in comparison to their parent cells, which can alter the expression of the TAK1 growth factor in recipient cells (Kogure *et al.*, 2011). Similarly, again working in cell culture, Chen *et al.* (2014) showed evidence that chemotherapeutic resistance between breast cancer cell lines was associated with trafficking of miRNAs within MVs. The *in vitro* repression of target mRNAs in endothelial cells by miRNAs trafficked from macrophages has also been demonstrated (Squadrito *et al.*, 2014). It is worth noting that episodic delivery of miRNAs in by MVs could contribute to considerable perturbation in gene expression.

miRNAs also have the potential to be trafficked in MVs between different species, though the extent and importance of this phenomenon is not yet known. Evidence to support the principle of this idea comes mostly from the study of immune biology. Proof of principle comes from the transfer and exchange of miRNAs between species, as shown in *in vitro* experiments in which nematode MVs containing miRNAs, YRNAs and an Argonaute protein were incubated with host mouse cells and showed suppression of gene expression of the genes targeted by the miRNAs (Buck *et al.*, 2014). The suggestion that this is also possible *in vivo* is supported by the evidence from the same study in which miRNAs from a filarial nematode were found in the blood serum of infected mice (Buck *et al.*, 2014).

The accumulating evidence shows that, in principle, miRNAs trafficked in MVs can serve as a mechanism of delivering genetic information. Increasing evidence supports the idea that MVs containing RNAs can be trafficked within individuals, between males and females (Corrigan *et al.*, 2014) and even between species (for example, between parasites and their hosts; Barteneva *et al.*, 2013; Buck *et al.*, 2014). Horizontal transfer of miRNAs via MVs between tumour cells is reported and may represent a mechanism for conferring chemoresistance (Chen *et al.*, 2014). miRNA-containing MVs have even been hypothesised as a mechanism for the transfer of epigenetic inheritance from germ line to soma (Sharma, 2014) and as potential contributors to mechanistic ageing (Weilner *et al.*, 2013).

The major outstanding question is whether these effects occur to any extent *in vivo* and the relative role of the different types of MVs in this process (Bobrie *et al.*, 2011). Even if the individual steps are all possible, we also need to know that all of them can occur efficiently in the same biological systems. The interesting possibility that MVs may contain biologically relevant sets of RNAs and other molecules that might act in concert has also hardly yet been considered.

### Conflict hypothesis for microguards and micromessengers

Above we reviewed evidence for miRNAs as microguards of the genome to prevent gene expression variation with the potential to result in fitness costs, and the potential role of miRNAs as messengers that can themselves perturb gene expression in potentially remote target cells. These new functions for miRNAs are likely to frequently place them at the heart of interactions between different parties with different fitness interests. For example, cancer cells and the antecedents from which they are derived, males and females, hosts and disease-causing organisms often have divergent evolutionary interests because of their lack of genetic relatedness. Genes in cancer cells increase in frequency via proliferation and have little interest in promoting the survival of their parent, non-cancerous host cells, from which they become less and less related over time as they accrue mutations. Similarly, in promiscuous mating systems males often gain by forcing their unrelated mates to invest more in the current reproductive bout than may be optimal for the long-term interests of females. Finally, disease-causing organisms gain fitness from proliferation within hosts and effective spread to the next host, which is may often not be in the best reproductive strategy for the host itself.

These evolutionary tensions between interacting parties can often be a potent force for driving evolutionary change (Chapman *et al.*, 2003a; Chapman, 2006). We explore these new ideas below and lay out a new hypothetical framework to highlight the role of conflict in shaping the evolution of microguards and micromessengers. Specifically we hypothesise that: (i) microguards may often function to dampen oscillations in gene frequency that arise precisely because of conflicts of interest; (ii) micromessengers may themselves be effective agents of conflict between different parties. It is clear that much more research is needed to test these hypotheses and we outline some of the approaches that could prove fruitful.

### Hypothesis: microguards evolve to dampen down conflicts of interest

In general, we expect gene expression variation resulting in significant fitness costs to be minimised within individuals, because of selection for efficient homoeostasis. However, as noted above, whenever individuals encounter perturbations to gene expression from exogenous sources, there is the potential for microguarding to increase fitness and therefore to be strongly selected. This is of key importance when specific host responses are being targeted, as is often the case when there are conflicts of interest. The reason why conflicts are of particular relevance in this context is that it is in these situations that perturbations to gene expression will occur, precisely because the different parties may be trying to manipulate the other and force maladaptive over-investment patterns (Table 1). Potentially deleterious gene expression variation and fluctuation is almost a necessary outcome of this interaction.

#### Within individuals

We expect microguarding to evolve as a general surveillance mechanism to buffer against normal variation in homoeostatic processes (Kim *et al.*, 2013). However, when the body encounters cells that have escaped homoeostatic control, for example, cancer cells that exhibit lower relatedness and increased heterogeneity in comparison with the rest of the cells in a tissue, then variation in mRNA levels is expected to increase and result in potentially maladaptive variation. Loss of homoeostatic control often accompanies the expression of mechanistic ageing. A recent review suggests that the release by cells of miRNA-filled MVs increases with age and that their resulting effects may be a contributory factor to the expression of proximate ageing (Weilner *et al.*, 2013). Many studies investigating evolutionary theories of ageing centred on mutation accumulation (Medawar, 1952) have sought supporting evidence by testing for increased variation in trait expression with age. It would be useful to test this idea further and to examine whether loss of mechanisms to regulate gene expression variation, potentially including the loss of microguarding, are contributory factors. One further idea is that in the context of ageing it is possible that over an individual's lifetime cells could lose the ability to regulate the release of MVs (Weilner *et al.*, 2013) or that target cells could lose the ability to buffer the effects of external perturbations such as those supplied by miRNAs delivered in MVs.

#### Between individuals of the same species

We predict that microguarding should be particularly prevalent in situations when there are conflicts of interest between the sexes. Such conflict has been well studied in fruit flies where it can be mediated via the actions of semen molecules transferred from males to females during mating (Chapman *et al.*, 2003a). As noted above, miRNAs via their microguarding functions can protect individuals against the deleterious effects of gene expression variation that occur in response to mating and the receipt of seminal fluid molecules (Gioti *et al.*, 2012; Fricke *et al.*, 2014). The finding that miR-279 and miR-317, which can potentially mediate buffering functions in females, are normally significantly downregulated following mating (Fricke *et al.*, 2014) suggests that males may currently have the upper hand in this conflict. Intriguingly, males potentially transfer miRNA-279 to females during mating, perhaps to give a ‘compensatory dose'. Lower levels of miR-279 and miR-317 act to suppress microguarding and elevate offspring production, which may be deleterious for females in the longer term. The significant fitness costs that often arise because of male–female interactions (Chapman *et al.*, 1995; 2003a; Arnqvist and Rowe, 2005; Chapman, 2006) appear to result, at least in part, due to the overactivation of reproductive processes in females. Hence mechanisms such as microguarding that can dampen and minimise such gene expression variation (Fricke *et al.* 2014) should be selected. That there is selectable genetic variation to minimise such costs is known (Wigby and Chapman, 2004) though the genetic basis of that response is as yet unclear.

Another context where there are often strong conflicts of interest between individuals of the same species are between parents and offspring. This can occur both before and after birth. For example, the maternal placenta–embryo axis is a site in which much conflict is predicted and indeed observed (Haig, 1993). This is because this site regulates the amount of provisioning that is directed towards the developing embryo from the maternal blood supply. Too little provisioning and the embryo suffers, too much and there may be costs to the mother (Haig, 1993). Of interest in terms of the involvement of miRNAs are recent findings that the placenta also releases MVs into the maternal blood supply during pregnancy (for example see, Sarker *et al.*, 2014). Furthermore, placental-derived MVs are reported to contain miRNAs (Ouyang *et al.*, 2014), which suggests a potential mechanism for the potential manipulation of the mother by the developing foetus.

#### Between individuals of different species

There are also often significant conflicts of interest between individuals of different species that directly interact and which may require buffering mechanisms to reduce costly outcomes. In the context of miRNA trafficking being a potential mechanism to mediate gene expression variation we are concerned with the intimate associations between hosts and the disease-causing organisms they harbour (Barteneva *et al.*, 2013). However, as yet, there seems little evidence for microguarding in this context.

Overall, we expect microguarding whenever there is the possibility of unregulated gene expression with negative fitness consequences (Table 1). Microguarding is expected to be prevalent particularly when there are marked differences in the evolutionary interests (that is, conflict) of the interacting parties involved (Table 1)—which is a testable prediction. Future research could usefully search for microguarding under these specific scenarios.

### Hypothesis: micromessengers are agents of conflict between different interacting parties

The potential for miRNAs to function as micromessengers to effect cell-to-cell communication at a distance represents considerable potential for the expression of conflicts of interest. For example, sexual conflict could be expressed if miRNAs delivered via MVs to a target cell in individuals of the opposite sex manipulated gene expression in a way that favours the fitness interests of the genes in the parent male cell over those of the recipient female cell. Below we suggest the various scenarios where conflicts could be expressed and describe evidence that is consistent with this hypothesis.

#### Within individuals

A potentially frequent example of conflicts of interest that could be mediated within individuals occurs between cancerous and non-cancerous cells within an individual. Cancerous cells that start to proliferate in an unregulated manner do so to further their own interests as they become genetically distinct from their surroundings (for example see Greaves, 2007). There are growing numbers of examples in which cancer cells show abnormal patterns of MV-associated miRNA export and trafficking. For example, there is correlative evidence that cancer cells can transfer chemoresistance horizontally between cells within hosts via miRNAs trafficked in MVs (Chen *et al.*, 2014), though more direct evidence would be welcome. Ohshima *et al.* (2010) also report the selective export of the miRNA *let-7* in MVs from a metastatic gastric cancer cell line, which was not observed in the control parental cell line. The export of the tumour-suppressive *let-7* from the cancer cells is proposed to maintain their tumourigenic state and increase their fitness through further proliferation. Clearly much more research is required to discover the frequency and fitness consequences to cancer cells and non cancer cells of miRNA trafficking.

#### Between individuals of the same species

There is evidence of MV transfer from males to females during mating (Corrigan *et al.*, 2014) and interactions of RNAs from those MVs with the female reproductive epithelium. The constituents of the MVs in this case are not yet known. But in principle, could be a mechanism for transferring genetic information from males to females during mating with the potential to exert biological effects (Figure 4). Though many more investigations are clearly needed, at least one miRNA, miR-279, which is implicated in microguarding, is expressed in the secondary cells of the male accessory glands (Figure 4) that produce the MVs described above. These cells can detach and be transferred to females during mating (Leiblich *et al.*, 2012), Furthermore, the MVs released from the secondary cells of the male accessory glands within the male reproductive tract can fuse with sperm, be transferred during mating and interact with the female reproductive tract (Corrigan *et al.*, 2014). Human MVs derived from the male prostate gland similarly fuse with sperm, suggesting that this phenomenon may be conserved across species. The receipt of human semen is known to induce the expression of immune genes in cells of the cervix (Sharkey *et al.*, 2007), though whether there is any involvement of miRNAs in this process is as yet unknown. We conclude that data to support the hypothesis of miRNAs trafficked by MVs in conflicts of interest between the sexes are currently scant and further studies to document the biological roles of miRNAs transferred in MVs are sorely needed.

#### Between individuals of different species

Hosts and the disease-causing organisms they harbour rarely have coincident fitness interests (Chapman, 2006) and so the increasing reports of disease phenotypes being mediated via the transfer of miRNAs in MVs is of interest of the conflict framework we propose here. As noted above, miRNAs from a filarial nematode have been detected in mouse serum and *in vitro* the targeted suppression of two genes in mouse cells incubated with miRNA-containing MVs derived from a gastrointestinal nematode have been reported (Buck *et al.*, 2014). In other cases, fungal pathogens have been observed to use small RNA transfer as a means to hijack and suppress host RNA silencing pathways, further expediting their invasion (Weiberg *etal.*, 2013). These findings illustrate a mechanism by which parasites might manipulate their hosts and also demonstrate RNA transfer between species. Such trafficking between species may even extend across even wider taxonomic distances. For example, plant-derived miRNAs in human and porcine breast milk have been identified *in silico* (Lukasik and Zielenkiewicz, 2014). However, the functional significance of these findings remains to be determined.

### Antecedents of microguarding

Variation in gene expression is likely an inevitable consequence of the mechanisms by which genes are regulated. However, whenever this variation is costly there will be selection for dampening mechanisms that could include microguards. In general we expect the tracking of gene expression by the mechanisms that control it to improve and become tighter over evolutionary time. These dynamics may also show differing patterns depending upon the relationship of the interacting parties, that is, the extent to which they are in evolutionary conflict. These ideas could in theory be tested *in vivo* in experimental evolution experiments via the creation of new pairings between miRNAs and targets by the creation of synthetic miRNAs or synthetic targets with engineered target seed sequences. This could be achieved by the genetic transformation of model organisms with engineered synthetic miRNAs or target sequences. It would also be of interest to explore whether there are qualitative differences in gene expression variation in different contexts. Future work will show whether microguarding provides a more or less tight control of gene expression variation to be deployed in certain contexts over others. When greater numbers of examples of microguards have been identified phylogenetic investigations of the evolution of microguards versus their target genes could also be informative in picking apart the evolutionary sequence of events involved.

## Summary

The study of RNA silencing, originating from experiments in animals such as *C. elegans* and *D. melanogaster* and plants such as *Petunia hybrida* and *Arabidopsis thaliana*, continues to uncover surprising and novel functions of regulatory RNAs. Microguarding—mediated by miRNAs—appears to be a fast-acting response to exogenously induced antagonistic gene fluctuations. This represents an additional facet of small RNA regulation in addition to the already known roles in long-term homoeostatic gene regulation and micromanaging (Bartel and Chen, 2004). The fitness costs of defective microguarding are predicted to result in accelerated ageing and increased disposition to infectious disease. Further studies are now needed to explore this concept and to identify further examples of microguarding. The reduction of ligation bias in genome-wide small RNA cloning for next generation sequencing is uncovering new classes of small RNA that were not previously considered (Xu *et al.*, 2015). We may uncover new miRNAs or other types of small RNA with a greater influence on microguarding. Our review has defined scenarios under which we expect microguarding to be prevalent, which may help focus the search for new examples. We also explored the significance of recent discoveries of trafficking of MVs containing miRNAs. Cells can release MVs whose contents exhibit some cell or tissue specificity, that can be delivered to specific target cells or tissues and that can in principle alter the expression of specific target genes in different tissues, individuals or even different species. In this way miRNAs can be part of a mechanism to both regulate and perturb gene expression.

We concluded by setting out two hypotheses in which we expect miRNAs to have significant roles in buffering against the effects of, or being the agents of, conflicts of interest. The scant evidence so far is consistent with these hypotheses but it is clear that many more data are needed before these ideas can be fully evaluated. Further exploration of the microguarding and micromessenger phenomena in model systems such as *D. melanogaster* and *C. elegans* could be especially informative. In a continuation of the evolutionary arms race between the sexes, males may transfer miRNAs or other small RNAs in their ejaculate during mating to disrupt or complement female microguarding that would otherwise jeopardise male mating success. The use of this system with its powerful experimental tractability may give clues to the significance of miRNA-containing MVs.

## Data archiving

There were no data to deposit.

## Figures and Tables

**Table 1:** Predictions for biotic scenarios in which microguarding will confer significant fitness benefits

*Source*	*Source of gene expression variation and potential for microguarding*
Within individuals	*Degree of developmental stability*: Mechanisms such as microguarding are expected to be important and efficient ways in which to carefully fine-tune developmental processes, ensuring the correct balance between developmental signals. Perturbations to these signals will disrupt normal development resulting in potentially significant fitness costs, selecting for any mechanisms that lower gene expression variation (Kim *et al.*, 2013).
Within individual conflict	*Selfish genetic elements such as driving genes and transposable elements*: relatively unregulated, and therefore potentially costly, gene expression perturbations may be common whenever the genetic causative agent involved lacks fitness interests that are coincident with that of the host in which it resides. Such a situation exists for selfish genetic elements that enhance their own transmission, sometimes at the expense of their host genome. Hence if mechanisms by which such elements excise, replicate and increase in frequency result in costly gene expression perturbation in the host, one outcome is selection for microguarding mechanisms. *Conflict within clones of cells within individuals:* conflict is expected between different cancer clones within individuals or between cancer clones and the hosts in which they reside. This has the potential to initiate unregulated gene expression variation and hence select for guarding mechanisms, as above.
Sexual conflict	*Conflict between males and females over reproductive decisions and shared reproductive traits*: conflicts over, for example, the pattern of traits (such as mating frequency or reproductive investment) that are shared between males and females are common. For example, males may gain fitness by manipulating their mates to invest more than optimal and vice versa. An example is observed in the transfer of semen components from male to female fruit flies during mating that cause females to significantly increase their reproductive investment by laying more eggs. Such effects can lead to costs and widespread gene expression changes in females. Our recent data suggest that miRNA expression in females can reduce the costs of these effects (Fricke *et al.*, 2014).
Sexual selection	*Competition within and between the sexes*: there is considerable variation in trait expression, and likely underlying gene expression, arising from male–male competition and from the expression of female mate choice. Costs arising from these competitions are also well known and could again select for gene expression dampening via microguards.
Parent offspring conflict	*Conflict between parents over the level of parental care*: conflict over the level of provisioning across the placenta from mother to developing arising from asymmetries of relatedness between mother, father and offspring are well known. The embryo may gain from manipulating the mother into investing more resources than is optimal for her. On the basis of recent findings there is the potential for miRNAs to be messengers, as well as potential guardians of this conflict.
Hybridisation	*Disruption of coadaptation via hybridisation*: matings between different populations or locally adapted genotypes could generate gene expression fluctuation because of perturbation of co-adapted gene networks. Such matings are often observed to result in unpredictable outcomes and potential costs (for example see Andres and Arnqvist 2001) and if sufficiently frequent could select for microguarding as a mechanism to buffer costs.
Host–microorganism conflict	*Conflicts between hosts and the microorganisms that reside within them*: the interests of hosts and their microbes are often not aligned. Success of the microorganisms may therefore often result in deleterious effects on the host, with selection for dampening mechanism such as microguarding.
